# Anonymity Preserving IoT-Based COVID-19 and Other Infectious Disease Contact Tracing Model

**DOI:** 10.1109/ACCESS.2020.3020513

**Published:** 2020-08-31

**Authors:** Lalit Garg, Emeka Chukwu, Nidal Nasser, Chinmay Chakraborty, Gaurav Garg

**Affiliations:** 1 Department of Computer Information System (CIS)Faculty of Information Communication Technology (ICT), University of Malta 37563 2080 Msida Malta; 2 College of EngineeringAlfaisal University 101686 Riyadh 50927 Saudi Arabia; 3 Department of Electronics and Communication EngineeringBirla Institute of Technology at Mesra Ranchi 835215 India; 4 ABV-Indian Institute of Information Technology and Management 121807 Gwalior 474015 India

**Keywords:** Contact tracing, RFID, IoT, blockchain, hospitals, telemedicine, digital health, privacy, COVID-19

## Abstract

Automated digital contact tracing is effective and efficient, and one of the non-pharmaceutical complementary approaches to mitigate and manage epidemics like Coronavirus disease 2019 (COVID-19). Despite the advantages of digital contact tracing, it is not widely used in the western world, including the US and Europe, due to strict privacy regulations and patient rights. We categorized the current approaches for contact tracing, namely: mobile service-provider-application, mobile network operators’ call detail, citizen-application, and IoT-based. Current measures for infection control and tracing do not include animals and moving objects like cars despite evidence that these moving objects can be infection carriers. In this article, we designed and presented a novel privacy anonymous IoT model. We presented an RFID proof-of-concept for this model. Our model leverages blockchain’s trust-oriented decentralization for on-chain data logging and retrieval. Our model solution will allow moving objects to receive or send notifications when they are close to a flagged, probable, or confirmed diseased case, or flagged place or object. We implemented and presented three prototype blockchain smart contracts for our model. We then simulated contract deployments and execution of functions. We presented the cost differentials. Our simulation results show less than one-second deployment and call time for smart contracts, though, in real life, it can be up to 25 seconds on Ethereum public blockchain. Our simulation results also show that it costs an average of $1.95 to deploy our prototype smart contracts, and an average of $0.34 to call our functions. Our model will make it easy to identify clusters of infection contacts and help deliver a notification for mass isolation while preserving individual privacy. Furthermore, it can be used to understand better human connectivity, model similar other infection spread network, and develop public policies to control the spread of COVID-19 while preparing for future epidemics.

## I. Introduction

Non-pharmacetical measures taken to contain outbreaks require the cooperation of data subjects. Transparency in how consents are obtained and how individual data is used continue to fuel mistrust amongst citizens [1]. The conflict between the right to know, censorship, and data privacy continues to grow. Traditional approaches to sharing information amongst healthcare stakeholders have been with the help of central intermediaries who facilitate care coordination [2]. Blockchain promises the trusted and secure decentralization of these intermediaries [3].

**FIGURE 1.**
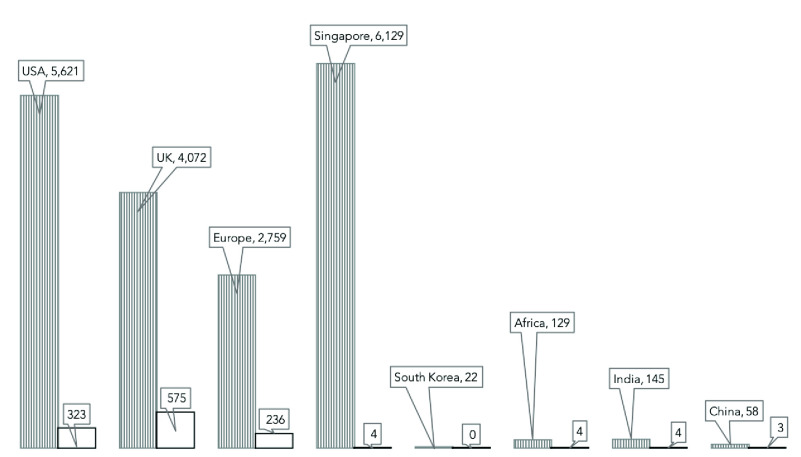
COVID-19 infections and deaths per million (June 2nd, 2020) [6].

The fear of massive surveillance and data misused has hampered voluntary and rapid containment of outbreaks like the Coronavirus disease 2019 (COVID-19) pandemic. The number of infected persons and deaths continue to grow, with wide disparity between jurisdictions with less-stringent privacy rules like China, Africa, Singapore, and South Korea, when compared with infections and deaths per population from the USA, Europe, and, the UK with stricter privacy control measures. Though other factors like the number of tests conducted, the accuracy of data, weather conditions and many more may have contributed. The rapidly evolving statistics from the pandemic as of June 2nd, 2020, in Figure 1 shows the number of infections and the number of deaths per million population [4], [5].

COVID-19 spreads mainly through person-to-person transmission and often through respiratory droplets and contact with surfaces or items contaminated [7]. Researchers believe that COVID-19 originated from animals [8]. Human to animal transmission is also possible [9].

### A. Study Rationale

Countries are set to reopen due to economic pressures, and concerns are high on how to sustain pandemic containment gains. We investigated how digital contact tracing is used as one of the many countermeasures against COVID-19. Based on our survey, initiatives around the world and current contact tracing solutions are centrally managed with attendant privacy concerns [10]. There are proposals to decentralize contact tracing data storage championed by Apple and Google, the leading mobile phone application providers [11]. These efforts have not enjoyed widespread public and political support. Moreover, these efforts replace one form of centralization with another. We hypothesize that citizen trust and privacy-concern may affect the voluntary adoption of potentially scalable solutions. Also, to our knowledge, there is no contact tracing solution targeted at moving objects or animals. One example of privacy fueled resistance to adoption is India’s Aarogy Setu application. It is now at the centre of a privacy controversy after initial launch successes [12].

Blockchain technology promises trust-oriented intermediation in healthcare [3]. This intermediation capability can help address citizen privacy concerns [3]. Therefore, we are proposing extending the current digital contact tracing solutions by updating anonymized contact proximity information to a blockchain as against traditional centralized government servers. In addition, we also propose the use of RFID transceiver to help track moving objects while logging anonymity preserving information on a blockchain.

### B. Paper Organization

The remaining sections of this article are organized as follows: Section II presents the definition of a case, and then contacts of a case. Section III presents brief literature on how the contact tracing concept is used for the current COVID19 response. Section IV explains the details of the proposed model, including system architecture, networks, and prototype. Section V discusses and interprets technical considerations and tradeoffs of our model while presenting its limitations. Finally, Section VI summarizes and concludes the paper and lay a foundation for future research.

### C. Research Questions (RQ)

The research questions answered by this study are:

• **RQ-1** *What are the current digital contact tracing strategies?*
• **RQ-2** *Which contact tracing approach or combination thereof can be used for moving objects?*
• **RQ-3** *What model can both scale and preserve privacy?*

## II. Contact Tracing and Warning Measure

**FIGURE 2.**
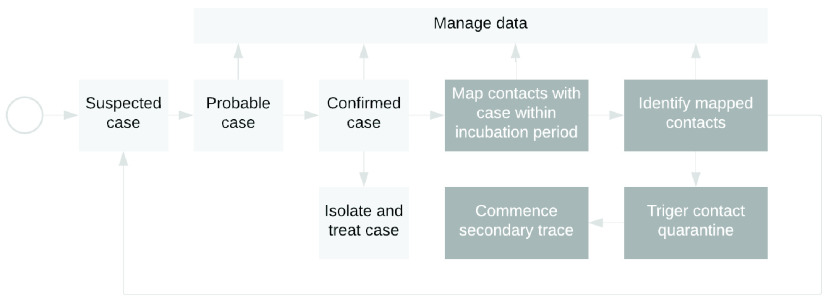
Disease case identification and contact tracing hypothetical workflow.

Non-pharmaceutical systematic contact tracing and enforcement of precautionary self-isolation is a key component of the global response [13]. A recent mathematical stochastic model shows that contact tracing can be useful if done within the first three months of COVID-19 or any outbreak [14]. The process often involves contact mapping, identification, isolation, confirmation, and treatment depicted in Figure 2. Contact tracing is the process presented by the dark shades in white print in the block diagram [15].

### A. Contact Definition

Contact tracing commences when a COVID-19 case is confirmed positive through a laboratory test. In traditional approaches, interviews will then be conducted with the case to ascertain their contacts up to 14 days following symptoms. The next step will be the identification of these contacts and initiating the contact tracing process [16]. The next two sections will define a COVID-19 case and their contacts following Figure 2 [15].

#### 1) COVID-19 Case Definition

**FIGURE 3.**
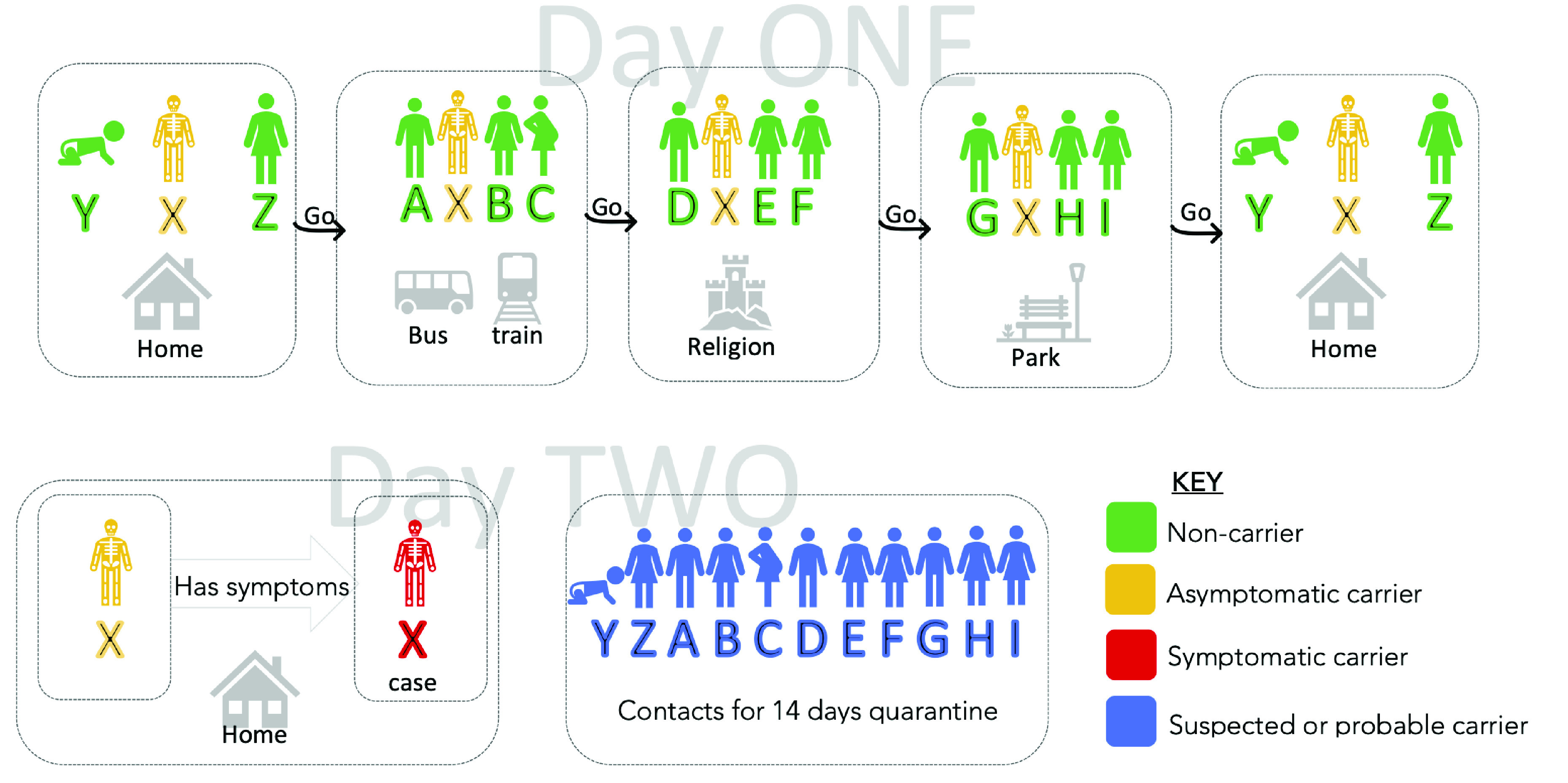
Illustrating a COVID-19 case and contacts.

The WHO and many country guidelines for managing COVID-19 give three definitions of a COVID-19 case [5]. In Figure 3, a case is represented by an individual X with mild symptoms of COVID-19. A suspected case has certain symptoms, in addition to travel or visit to certain locations. A probable case is a suspected case with an inconclusive laboratory test. A confirmed case is generally based on a positive result from a laboratory test.

#### 2) COVID-19 Contact Definition

Similarly, a contact, as defined in [15], can be one of the following:

• “Having face-to-face contact with a COVID-19 patient within 1 meter and for greater than 15 minutes”;
• “Providing direct care for patients with COVID-19 disease without using proper personal protective equipment”;
• “Staying in the same close environment as a COVID-19 patient (including sharing a workplace, classroom or household or being at the same gathering) for any amount of time”;
• “Travelling in close proximity with (that is, within 2 m separation from) a COVID-19 patient in any kind of conveyance”;
• “and other situations, as indicated by local risk assessment.”

**FIGURE 4.**
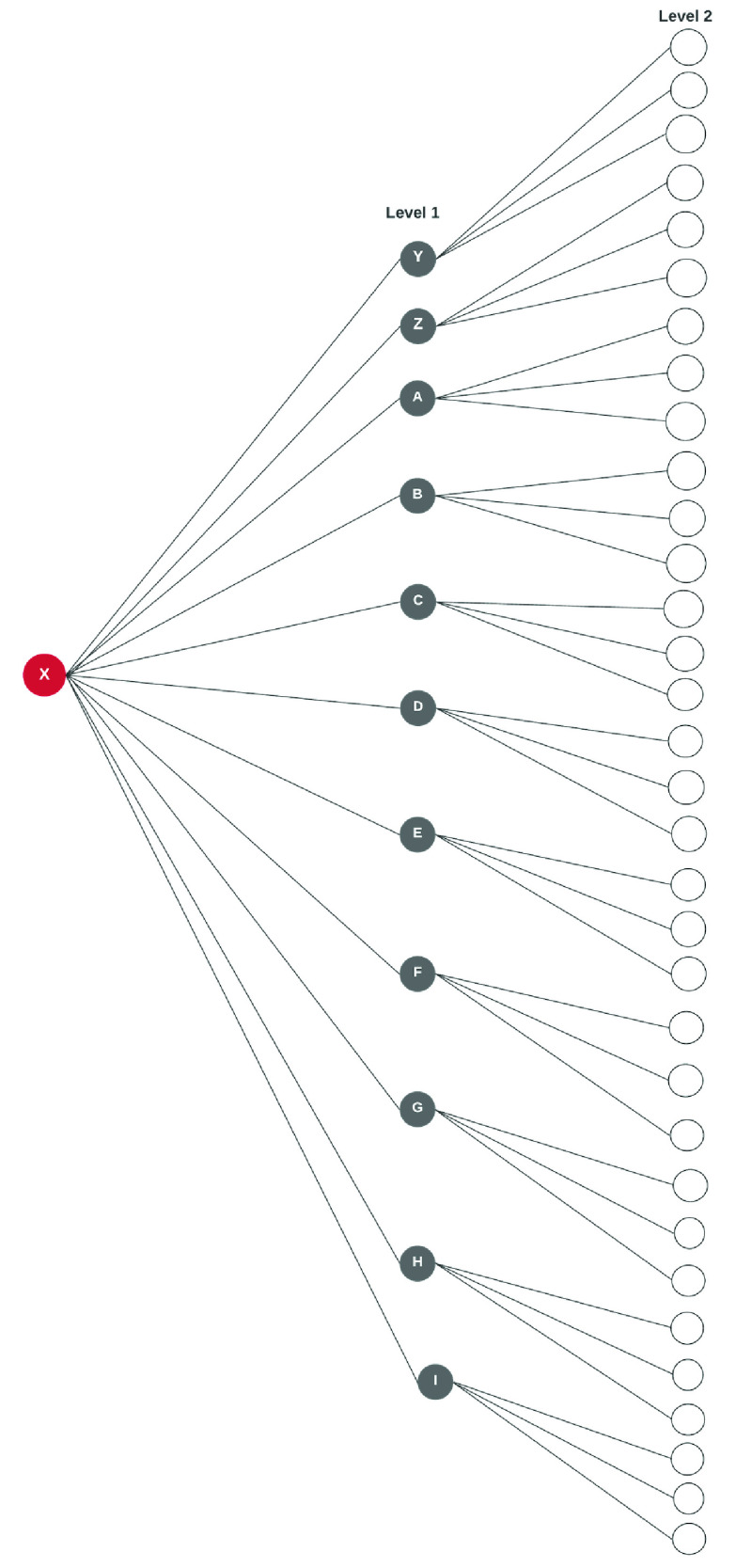
Exponential contacts and infection possibilities from index case.

We use Figure 3 to illustrate a contact. If case X prior to confirmation (or who is asymptomatic) visited a park and a place of worship and had contacts as in the figure on day one. It follows that individuals A,B,C,D,E,F,G,H,I,Y,Z are all contacts of case X before symptoms or test confirmation. To effectively curb the spread, a multi-level contact tracing is inevitable globally. This approach will ultimately aid the easing of restrictions and lockdowns. As shown in Figure 4, Our case X from Figure 3 can potentially infect hundreds more in level 2.

## III. Contact Tracing Literature

Traditional contact tracing and notification rely on the ability of contacts to know, recall, and have the names and mobile numbers of the persons they have been in contact with during interview [16]. This may not be practicable, or the contact may not be corporative. Our exploratory internet search shows that current digital approaches to contact tracing can be grouped according to data sources into service provider mobile app, citizen mobile app, Call Detail Records (CDR), and hardware enabled. These solutions, as shown in Table 1, are discussed in detail in the subsections that follow.

**TABLE 1:** Comparing the State of Different Contact Tracing Approaches

Contact tracing solution	Country	User	Data input	Data custodian	Network
GSM CDR [21]	Sierra Leone	Telco	CDR	Govt.	Telcom
SA-MoH CDR [22]	Saudi Arabia	Govt.	CDR	Govt.	Cellular
Tabaud [27]	Saudi Arabia	Govt.	Citizen-app	Govt.	Bluetooth, WiFi, Cellular
TraceTogether [28]	Singapore	Citizen	Citizen-app	Govt.	Bluetooth, WiFi, Cellular
Aarogy Setu [29]	India	Citizen	Citizen-app	Govt.	Bluetooth, WiFi, Cellular
MA call center [23]	USA	Call agents	Provider-call, CDR	Govt.	Telephone
Health code [30]	China	Citizen	Citizen-app	Govt.	WiFi, Cellular, & Bluetooth
Contact tracers [20]	China	Provider	Provider-app	Govt.	NA
Big data analysis [31]	China	Govt.	IoT	Govt.	WiFi
ProteGO [32]	Poland	Citizen	Citizen-app	Govt.	Bluetooth, WiFi & Cellular
Wrist band [33]	Hong Kong	Citizen	IoT, Citizen-app	Govt.	Bluetooth, IoT, WiFi, Cellular
Our model	Global	All	IoT, Citizen-app, provider-app	Blockchain	Bluetooth, IoT, WiFi, Cellular

### A. Service Provider Mobile App

A service provider application is a digital application used by healthcare service providers to track the contacts of a person with a confirmed case of infection. One such application is an electronic form with the CommCare smartphone application as one example [18]. The government of Sierra Leone introduced the CommCare application for COVID-19 contact tracing [18]. A similar system was used for Ebola contact tracing, and the proof-of-concept study found that despite many challenges, the use of the application was evaluated and found to improve completeness, accuracy, and storage of data [17]. In response to COVID-19, China worked with WHO at the early stage of disease onset to use form-based contact tracing completed by field agents [19].

### B. Call Detail Records Analysis

According to GSMA intelligence, there are over six billion connected subscriber identification Numbers (SIM) globally. The mobile phone is ubiquitous even for remote locations with limited internet access, and it is possible to mine subscriber location data from base-station triangulation.

Authors in [36] describe how mobile phone call detail data about population movement patterns can be used for early COVID-19 cluster identification and notification.

#### 1) Sierra Leone Cdr Analysis

The International Telecommunications Union (ITU) leveraged CDR to support the contact tracing efforts aimed to curb the spread of EVD in Sierra Leone, along with neighboring countries Liberia and Guinea [20]. The call detail record is information available from a telecommunication-provider equipment or the communications-exchange-provider equipment detailing mobile communication and mobility activities.

The CDR data set covered a period June, and July 2015 covering 1.8 billion call records with a file size of 207GB spread across Africell, Airtel, and Smart (only 272MB) [20]. Human movement analysis was conducted at the city-to-city scale and transnational boundary movements between Sierra Leone, Guinea, and Liberia. The city-to-city scale analysis shows a strong correlation between mobile phone users and the actual population movement. This information was then leveraged by authorities to manage containment—the transnational move allowed for a better understanding of movement patterns during the outbreak. Similarly, the Ministry of Health in Saudi Arabia recently passed an emergency law to use the same strategy for COVID-19 contact tracing [21]. These reports highlighted significant limitations and difficulties:

• Privacy concern – Constant conflict between using innovation and rights to privacy
• Accuracy – Almost all CDR data use base tower locations to infer device location with accuracy between 50 and 300 meters.
• Availability of data – Operators may not collect some data points
• Data discontinuity

Based on [20], MNOs will be requested to provide a dataset with the following data elements.

• Device unique International Mobile Equipment Identity (IMEI) for calling and called parties irreversibly encrypted using hash functions.
• International Mobile Subscriber Identity (IMSI) for calling and called parties irreversibly encrypted using hash functions.
• The timestamp of call-start and call-end in YYYY-MM-DD:mm: ss format.
• The base station of the identity of called and calling parties identifying LAC, cell identity, longitude, and latitude.
• Mobile phone number of called and calling parties irreversibly encrypted using hash functions.
• Activity type for called and calling party classified into either voice, SMS, or data. The researchers further classified the data component into 2G, 3G, and LTE.

#### 2) MIT Call Center

The government of the state of Massachusetts announced it is launching the ’first’ contact tracing system in the US [22]. The system will be using a network of virtual 1000 call assistants to follow-up with contacts of any COVID-19 positive patient in the state. Similarly, as part of a two trillion dollar COVID-19 stimulus package passed by US congress, it is estimated that the US Center for Disease Control (CDC) will have $ 500 million for ’surveillance’ purposes and report on progress monthly [23]. The details of the CDC’s strategy will be available at their first report, which is not due at the time of this writing.

### C. Citizen Mobile Application

Citizen-application for contact tracing is by far the most adopted by countries worldwide with Google and Apple launching an API after initial joint public announcements on April 10th, 2020 [24]. As in Figure 3, we here discuss the workflow of most citizen application. They come in two major categories: Bluetooth tracked, and Global Positioning System (GPS) tracked. If the individual X within 14 days has the application installed, it is possible to use GPS (or Bluetooth) co-localizations with other persons using the app that are in close proximity. If on day 1, the individual X had contacts with persons at home, train or bus, and work, and all persons he had contact with, had the health code application installed. Then when X shows symptoms on day two and requests a test, and if the test returns positive, then all his contacts in the last 14 days are notified to self-isolate. Recent examples of citizen-facing application are shown next.

#### 1) EU PEPP-PT

The European Union (EU) funded a report “Mobile application to support contact tracing in the EU’s fight against COVID-19, Common EU toolbox for member state” on April 15th, 2020 [25]. Several EU countries are investigating the use of the Pan-European Privacy-Preserving Tracing (PEPP-PT) citizen facing application [25]. Table 1 highlights a few cases that inform the PEPP-PT overall strategy.

#### 2) TraceTogether

The Singapore government launched the TraceTogether app on March 20th, 2020, and within a week has recorded over half a million downloads. The app designed to help users know when they may be in close contact with someone with the COVID-19. Phones that have the app exchange short distance Bluetooth signals when they are near. This information is stored for 21 days and destroyed afterwards. This information has the location timeline of the phone in addition to other physical and digital logs collected [27].

#### 3) TABAUD

The Saudi Data and Artificial Intelligence Authority (SDAIA) launched an open-source application that, when downloaded, can warn users of Coronavirus case. The app works by taking location information from a user’s phone and comparing it with what is available on the ministry’s server and can use the collected and arrived intelligent information to alert the user of a potential nearby suspected, probable, or confirmed COVID-19 patient exposure [26]. The development and deployment of this application was an effort that followed the initial use of telegram to publish the movement of people infected with the virus on the ministry of health website.

#### 4) Aarogya Setu

The Indian government launched the Aarogya mobile application to help alert citizens when they have been in close contact with a confirmed COVID-19 patient or their primary contact [28]. In the first three days of launch, an estimated three million downloads were recorded, signaling the acceptance and possible success. Though in a country of 1.3billion people, time is needed to evaluate its success. Besides, India is one of the countries that has been able to keep its number of cases low despite its large population.

#### 5) Health Code

The Health Code contact tracing system was deployed extensively and mandatorily in Wuhan China [29]. Due to almost universal smartphone ownership in China, and the government’s high surveillance architecture, it was possible to achieve compliance. Residents of Wuhan and the industrial area of China are now mandatorily required to download a contact tracing/tracking application on their smartphones. According to Olivia Zhang, when accessing essential services like subway, station, an attendant with a banner with the inscription, “Please wear a mask throughout your trip. Do not get close to others. Scan the code before you get off the train.” Scanning the barcode on the poster triggers the passenger’s Health Code app. A green code and part of the passenger’s identity card number appears on the screen, and the guard then allows access [29].

### D. Hardware Solutions

Governments with extensive digital solutions network are using hardware-enabled solutions to measure the proximity of contacts to a case and the duration of the contact. Our survey shows a few of these measures.

#### 1) MagicBand-ESQUE Wristband

As early as March 2020, the government of Hong Kong announced plans to have anyone arriving in the country wear a mandatory wristband. The wristband will be used to enforced quarantine by capturing changes in location. The Chief Information Officer confirmed it would not capture location information, but changes in location to minimize treat to wearer’s privacy [30].

#### 2) CCTV Video

China is using the power of big data and artificial intelligence with a network of cameras and thermal scanner sensors to combat COVID-19 and maybe future epidemics [38]. The Chinese authorities are believed to be using facial recognition software from their camera networks to analyze the big data and come up with contacts and contacts-of-contact.

## IV. The Proposed Model

We are proposing an Internet of Things (IoT) hardware model that captures information on movements and contact of objects. Our model ensures that this is anonymously executed until holders have tested positive for an infection disease like COVID-19. As a proof-of-concept, we will use a passive RFID transceiver for the IoT component hardware. Animals and individuals can wear a passive RFID tag without having mobile phones on them. In order to guarantee use, it is best used to access service while accumulating points. To best of our knowledge, this is the first solution proposing IoT and specifically RFID for anonymized RFID contact tracing of infection spread. Our model also proposed the use of blockchain for data storage to ensure that privacy is preserved through distributed ownership and control of stored data. The readings are taken by the RFID reader, which can be in a building or a power device like vehicles. The captured proximity information is stored on the relevant Smart Contract (SC).

### A. Architecture and Network

**FIGURE 5.**
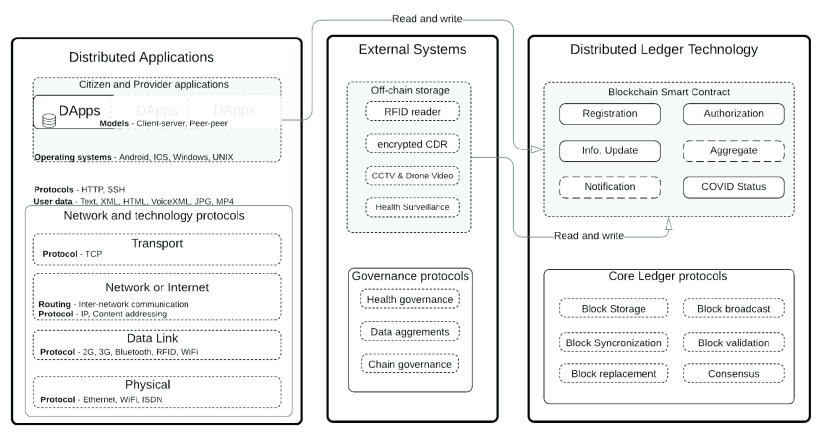
Model base system components and protocols.

The architecture for our proposed model, which is as in Figure 5, shows three component parts with their protocols. The Distributed Applications (DApp), the Distributed Ledger Technology (DLT) and the External Systems. The DApp is the frontend where users interface with the architecture either from a citizen mobile application or a health system provider application. The DLT is the backend in the model’s architecture using the client-server architecture paradigm description model. External systems can be systems capable of storing additional information for other purpose, and only allow information linkage on demand. The technology, healthcare governance or blockchain ledger protocol all determine how a system is implemented in production. We present the generic checkboxes for universal implementation.

**FIGURE 6.**
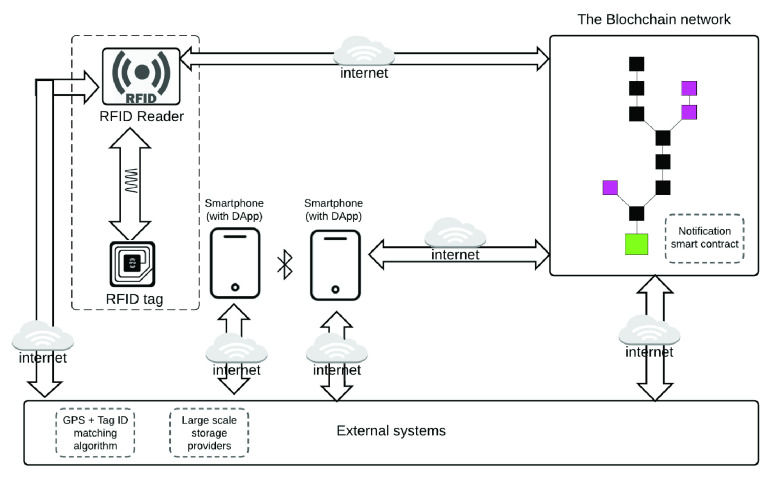
RFID device data flow diagram to the blockchain.

Our visual architecture model represented in Figure 6 shows how data flows from RFID tag to the reader to the blockchain. It also shows how proximity data collected by citizen-application contact tracer flows to the blockchain over the internet. Citizens apps linked to our model can generate, manage, and store their cryptographic keys using their compliant application of choice. The blockchain infrastructure helps log anonymized mobile device or RFID tag information on a public blockchain. Logged information can be used to message contacts if a citizen with the mobile device or RFID tag becomes a COVID-19 or other infectious disease confirmed case. Each component of our model is described in detail in the subsections that follow.

### B. Distributed Applications

**FIGURE 7.**
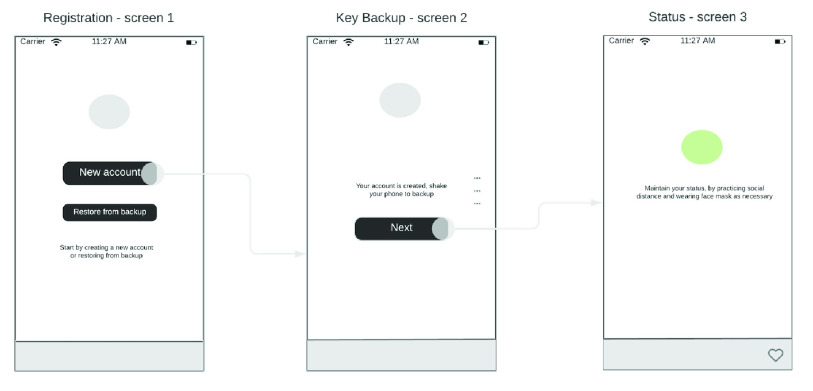
Registration flow user interface view.

**FIGURE 8.**
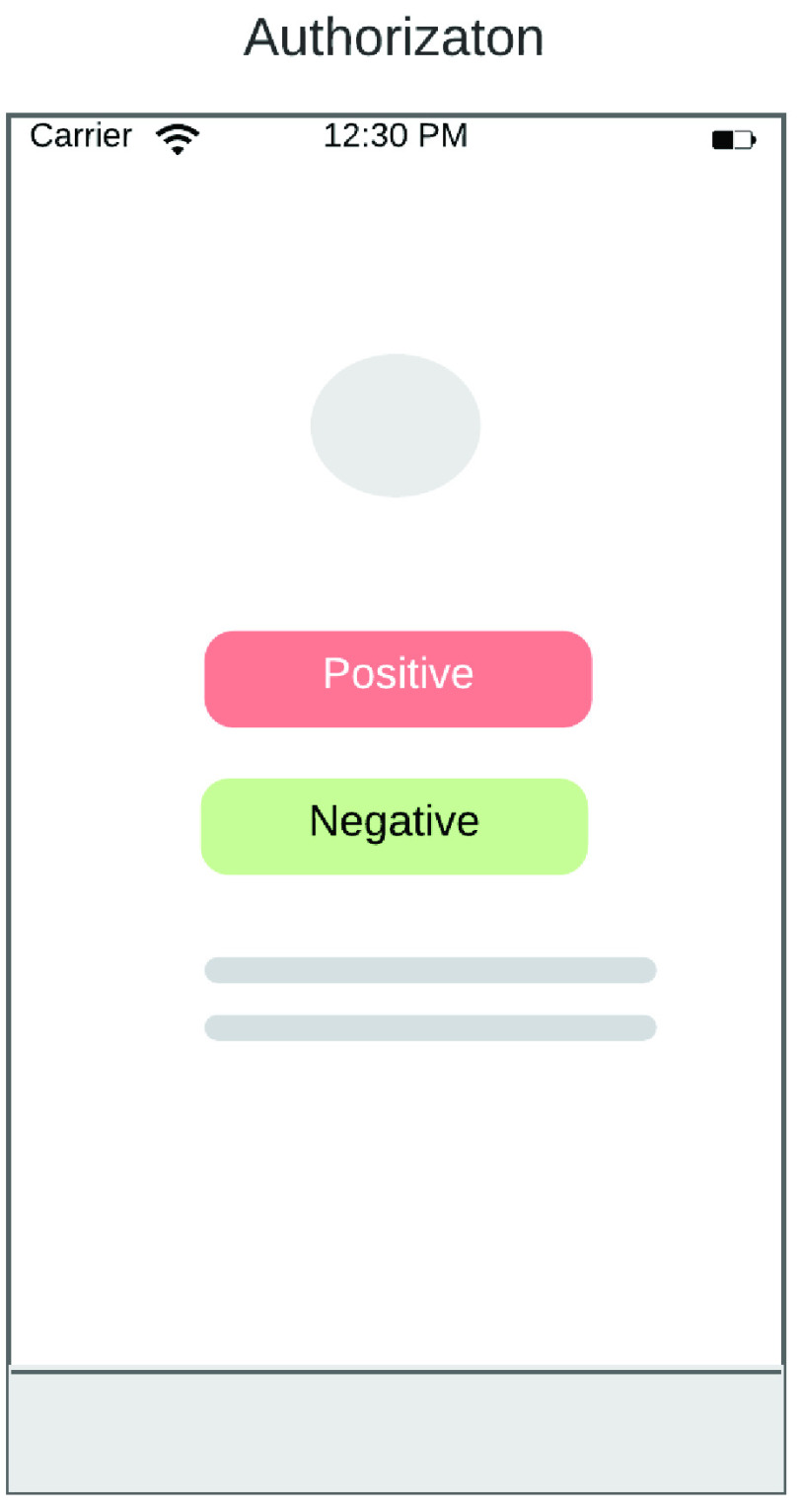
Authorization smart contract flow user interface view.

Our survey show there are two broad approaches for implementing citizen-facing distributed application (DApps). One is ad hoc phone-to-phone mesh network topology where each node is connected to every other node through some channel, notably Bluetooth. They share location information directly without the need for a hub. An ad hoc network can be configured to use WiFi Direct or Bluetooth protocols as the channels. Similarly, DApps simply capture the GPS location, which is more accurate and use the location and time of capture to determine close contacts and share notifications as necessary. The notification will be based on the application’s message notification interface over HTTP protocol. The DApps user interface for calling and updating data on the blockchain smart contract is shown in the mockup interfacing in Figure 7 and Figure 8.

### C. RFID Interfaces

Our system architecture facilitates connection to the external RFID reader to the blockchain via the internet. The location of an RFID tag and receiver in our architecture is as described in Figure 6. We propose a read-only passive RFID tag due to its low cost and low power. The tag will have a unique factory serial number which will be used as an access mechanism to the location update chain. The RFID tag simply logs its serial number information with the receiver when interrogated by the receiver. Our design is proposing that the receiver be situated at strategic locations and powered with utility power supply or battery. Moving objects like cars will have secondary receivers for tracking moving objects and their duration in certain locations. Similarly, secondary receivers like cars will have tags which can be read at strategic locations (e.g., toll gates). This will allow for location triangulation in the event of an outbreak. To mitigate the challenge of receivers’ sensitivity, they may be placed at entryway to strategic locations. It could be designed as an access token as already used for doorway access in many institutions.

The RFID receiver will interrogate, identify and also compute the relative distance of the tag to the receiver and use the information to triangulate the proximity of the tag. Information (proximity, tag serial number, and timestamp). The serial number of a tag can be read when a holder or their animal or other moving object tests positive or has been in contact with a confirmed case. A positive flag for an RFID tag holder will raise a notification that anyone subscribed to the blockchain can receive if they have been in contact.

### D. DLT and Smart Contract Layer

DLT is a technology implementation where a ledger is distributed across multiple computing devices, and often over multiple geographic domains. Blockchain is one type of DLT, that uses the peer-2-peer network model to connect participants in the network [31]. A blockchain as the name implies is a growing list of blocks representing transactions and other metadata.

Participants called nodes use the “gossip” protocol to propagate and verify transactions [32]. Blockchain is often an append-only ledger that is difficult to modify under normal conditions. The rules for how messages (or transactions) representing state changes are appended to the ledger is cryptographically defined for any given blockchain. This rule is referred to as consensus algorithm, and it can proof-based or vote-based [3]. The consensus mechanism is implemented as part of the core layer of any blockchain implementation.

A smart contract is a self-executing code that mimic’s traditional contracts only that it is code enabled. It is used in blockchain networks to extend blockchain capability by enforcing trust arrangements. For example, a smart contract can be configured to issue payments on-behalf of parties in a contract. Smart contracts were first introduced on Ethereum, one of the two main public blockchain network. Our model has implemented three smart contracts as a prototype to tests our model. They are the registration, update, and authorization smart contracts. They were implemented and tested in the Remix Integrated Development Environment (IDE). We did not implement the identification and notification contract in our prototype. The code snippets are available on github site [33].

#### 1) Registration Smart Contract

At registration, no information is collected except the generation of both public and private key pair by the application, which is done on the device. One key is registered as the public key on the blockchain through events for accessing the smart contracts, the other is stored on the device, with an option to backup. The registration process flow user interface front-end is as in Figure 7.

When a ’new account’ is tapped, two keys are generated, and one is randomly designated as the public key and flagged as the address of the generating entity. This smart contract is generated by either a user with an application as in Figure 6, or an RFID reader unbehalf of a passive RFID tag. The solidity code is in *Algorithm 1**, line 1 - 21*

Algorithm 1: **Registration.sol Solidity Smart Contrac**
Input: Serial number of tag or IMEI of phone “serial_imei”; $S=\{s_{1}, s_{2}, \ldots, s_{n}\}$
Output: The timestamp and the hash of serial or phone IMEI, “pub” (T, H); $H=\{h_{1}, h_{2}, \ldots, h_{n}\}$, $T=\{t_{1}, t_{2}, \ldots, t_{n}\}$
1 pragma solidity 0.4.25;
2 contract registration {
3 uint private serial_imei;
4 bytes32 public pub;
5 uint timestamp;
6 event register(
7 uint timestamp,
8 bytes32 pub
9 );
10 function captureRFID(uint _s_i) public{
11 timestamp = now;
12 serial_imei = _s_i;
13 pub = sha256(abi.encode(serial_imei));
14 emit register(timestamp, pub);
15 }
16 function enroll() public {
17 timestamp = now;
18 pub = sha256(abi.encode(msg.sender));
19 emit register(timestamp, pub);
20 }
21 }

The *registration.sol* smart contract uses solidity version 0.4.25 and above, with a contract name ’registration’. The ’pub’ variable can be used as a hash of the RFID tag or a hash of a mobile device’s IMEI number. The timestamp is generated at the time of calling the smart contract. The functions *captureRFID()* perform the functions of capturing the serial number of the RFID tag or the IMEI of the phone. The function takes the argument of the serial number captured by the reader. Similarly, the function *enroll()* is called when a mobile phone of citizen application users wishes to enrol to the blockchain.

#### 2) Update Smart Contract

The *update.sol* smart contract pushes information onto the blockchain from the mobile device or the RFID receiver after initial registration. It has two methods called depending on the device type. If an RFID receiver is accessing the blockchain on-behalf of a tag on an animal, then the *log serial()* function will be called with the parameter being the *serial number* of the tag. If a phone is calling the smart contract over the internet, the phone will call the *log imei()* function providing the *imei* number of the phone and a parameter ${H}$ which is the concatenation of all Bluetooth serials connected to the phone. See the solidity code in *Algorithm 2**, line 1 - 34*

Algorithm 2: **Update.sol Solidity Smart Contrac**
Input: Serial number of tag $S=\{s_{1}, s_{2}, \ldots, s_{n}\}$ or IMEI of phone $I=\{i_{1}, i_{2}, \ldots, i_{n}\}$
Output: The timestamp and the hash of serial or phone IMEI (T, H); $H=\{h_{1}, h_{2}, \ldots, h_{n}\}$, $T=\{t_{1}, t_{2}, \ldots, t_{n}\}$
1 pragma solidity 0.4.25;
2 contract update {
3 uint private serial_imei;
4 uint timestamp;
5 bytes32 private H;
6 bytes32 private pub;
7 event update_serial(
8 uint timestamp,
9 bytes32 pub
10 );
11 event update_imei(
12 uint timestamp,
13 bytes32 pub,
14 bytes32 H
15 );
16 function log_serial(uint _s_i)
17 public{
18 serial_imei = _s_i;
19 pub =
20 sha256(abi.encode(serial_imei));
21 timestamp = now;
22 emit update_serial(timestamp, pub);
23 }
24 function
25 log_imei(uint _s_i, uint _H)
26 public{
27 serial_imei = _s_i;
28 H = sha256(abi.encode(_H));
29 pub =
30 sha256(abi.encode(serial_imei));
31 timestamp = now;
32 emit update_imei(timestamp, H, pub);
33 }
34 }

This will only work for mobile devices participating and using compatible mobile applications. The RFID receiver or the phone repeats this process every 5 minutes. The calling of this contract happens in the background without the user intervention, as consent would have been provided at signup.

To reduce excessive data and battery usage for the phone, capture information about location changes every ten minutes. Upload to the blockchain will also be every 20 minutes for the same reason. A user’s identification on the blockchain is the public key which remembers its generator and other public key peers and records of contact. The model application does not track the actual location of the device, which is a proxy of the location of the person. The application will only track the distance between application users. The logging would commence as soon as the distance is less than two meters even if the connections happened at a further distance. This information will be used to obtain the duration of connection with anyone or device. The distance is tracked by the devices, and used to log serial number, but not stored.

The RFID tag does not have the capability to connect to other devices; its contact with similar other devices will be determined by timestamp information from the same received that coincide.

#### 3) Authorisation Smart Contract

The *authorization.sol* smart contract as in *Algorithm 3**, line 1 - 31* is the contract that is configured on a device lavailable to the health authorities that can mark a user close to a case as probable, suspected or confirmed case.

Algorithm 3: **Authorization.sol Solidity Smart Contrac**
Input: Covid status event log
Output: The timestamp and the hash of serial or phone IMEI, and status (T, H, CS); $T=\{t_{1}, t_{2}, \ldots, t_{n}\}$, $H=\{h_{1}, h_{2}, \ldots, h_{n}\}$, $CS=\{cs_{1}, cs_{2}, \ldots, cs_{n}\}$
1 pragma solidity 0.4.25;
2 contract authorization {
3 uint private serial_imei;
4 uint timestamp;
5 address pub_key;
6 uint covid_status = 0;
7 event update_positive(
8 uint timestamp,
9 address pub_key,
10 uint covid_status
11 );
12 event update_negative(
13 uint timestamp,
14 address pub_key,
15 uint covid_status
16 );
17 function log_positive (address _pub_key) public{
18 pub_key = _pub_key;
19 covid_status = 1;
20 timestamp = now;
21 emit update_positive
22 (timestamp, pub_key, covid_status);
23 }
24 function log_negative(address _pub_key) public{
25 pub_key = _pub_key;
26 covid_status = 0;
27 timestamp = now;
28 emit update_negative
29 (timestamp, pub_key, covid_status);
30 }
31 }

In the prototype contract, we have shown how a variable *covid status* can have a default value of 0. And then depending on the status of the device holder (phone or RFID tag), the healthcare provider can tap a button when in close proximity of the user and their device in an isolated room. In this prototype, there are two buttons that can be used to trigger these contracts as shown in Figure 8.

The button will trigger the *update positive* smart contract which, will set the flag *covid status* to 1, indicating the device holder is now positive. From that time on, any contact with the person is logged as positive. Conversely, when the patient recovers, the same process will be used to call the *update negative* contract to set the contact status.

## V. The Model Implementation

We implemented our model by using Remix IDE with three node addresses on one computer with specification as Mac book pro 2.5 GHz, 16GB, 500GB. The Remix ID test environment is a browser-based testing environment for the Ethereum blockchain network. Remix runs on Google Chrome browser. Ethereum has the largest community of public blockchain and is widely used particularly for the smart contracts feature [34]. The Ethereum blockchain uses a gas fee to measure the cost of transactions on the blockchain.

**FIGURE 9.**
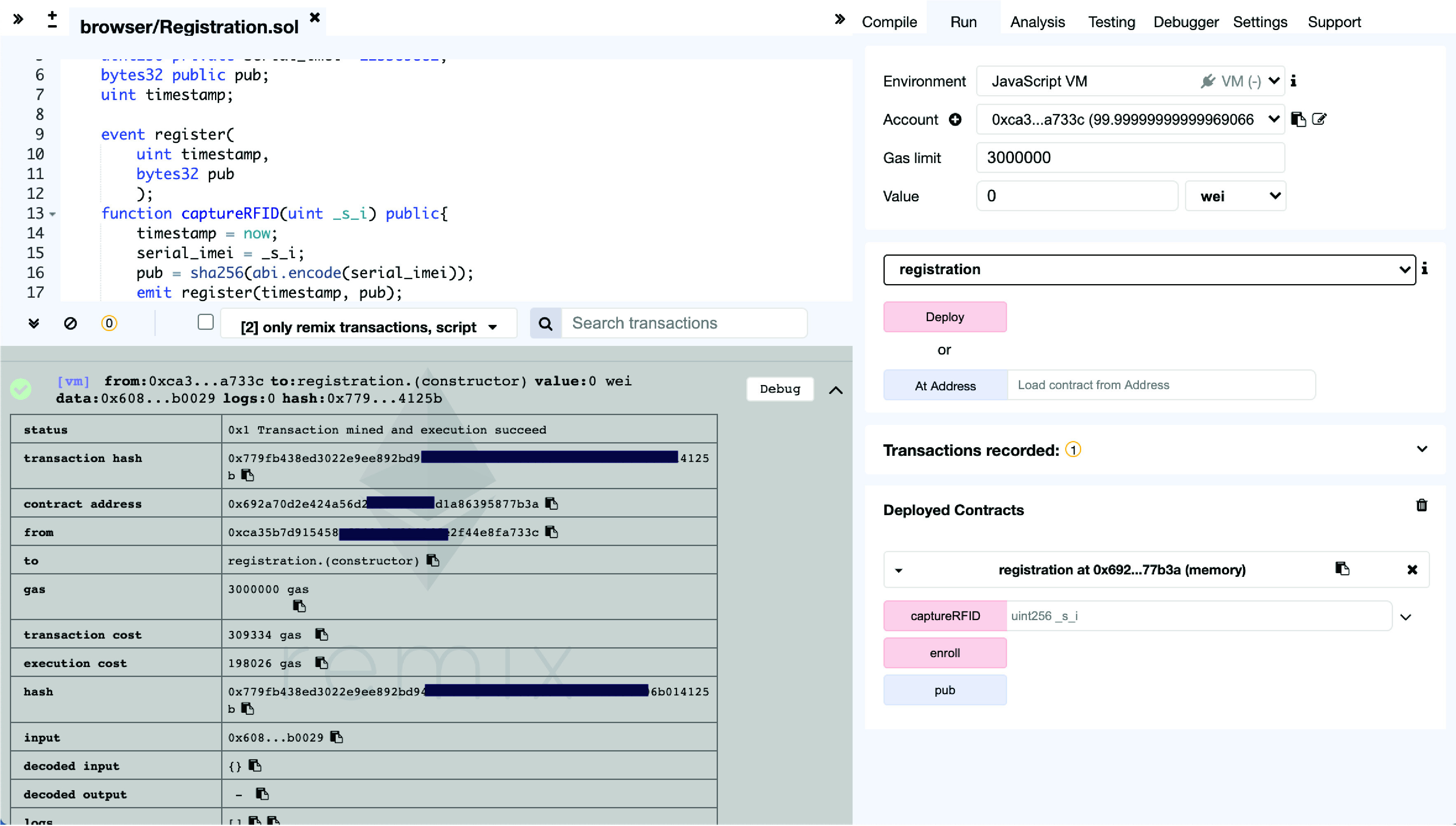
Remix IDE simulation interface.

We configured the three smart contracts as *.sol* files that can be consumed by the Remix IDE through the chrome browser. We implemented them on the Remix interface for our simulation as shown in Figure 9. We started by deploying the smart contract, then measure the gas execution cost. We then ran equivalents of the input data for each function in each of the three smart contracts. The results of our tests are shown in Table 2. In addition, we also used [35] to determine the estimated actual fiat value in US dollar using the current costing from the Ethereum pricing calculator [35].

**TABLE 2:** Simulation Results Showing Gas Consumption and Transaction Fees

Contract	Activity	Gas used	Transaction fee ETH	Transaction fee FIAT
Registration SC	deploying Contract	198,026	0.0083171	$ 2.02
	calling CaptureRFID function	13,750	0.0005775	$0.14
	calling Enrol function	43,267	0.0018172	$ 0.44
Information Update SC	deploying Contract	228,457	0.0095952	$2.33
	calling log serial function	8,906	0.0003741	$ 0.09
	calling log imei function	65,578	0.0027543	$0.67
Authorization SC	deploying Contract	146,596	0.006157	$ 1.50
	calling log positive function	27,986	0.0011754	$0.29
	calling log negative function	42,964	0.0018045	$ 0.44

### A. Simulation Results

Our simulation results show that though these transactions take less than a second to complete on Remix, on the public Ethereum network, it will take 25 seconds. We found that it costs an average of $1.95 to deploy our smart contracts and almost $0.34 to call and update functions in those smart contracts. Deployment is done once when the smart contract is set up. Similarly, devices like smartphones or RFID transceivers can call smart contract functions to enroll, update, or authorize status transactions.

## VI. Discussions and Limitations

In this section, we discuss our research findings and limitations of our study.

### A. Discussions

The discussions are grouped by the research questions.

### B. Research Question-1

*What are the current digital contact tracing strategies?*

Our survey has shown that while there are many different approaches to contact tracing, they can be grouped into four main categories. These categories presented in section three are service-provider-facing mobile application based contact tracing, which is an improvement of the traditional public health paper contact tracing; Analysis of call detail information from mobile network operators; Citizen-facing mobile application that makes use of an ad hoc mesh network to measure location and proximity information; And then hardware-based solutions like a wrist band and video surveillance based solutions.

### C. Research Question-2

*Which contact tracing approach or combination thereof can be used for moving objects?*

Despite the many approaches deployed for contact tracing, to our knowledge, no measure is in place for tracing some moving objects like animals or cars. We consider the RFID transceiver which we propose here the most effective and implementable mechanism for tracking moving objects. Wristband Bluetooth-based contact tracing can equally help, but due to technology sophistication, the cost can be prohibitive to implementing this scheme at scale. In addition, the need to continually charge and power Bluetooth solution increases the cost of operation of such a solution.

Our proposal is to have the RFID tags, and their readers store hashed information on the blockchain along with relevant data elements. While the case information is not linkable on the blockchain, users who have met the contact criteria set on the blockchain smart contract will receive a notification on their application with the current status of either yellow or green. If yellow, they have been in contact with a case and should follow the protocol to either self-isolate or go for a test. If the application show green, it means they have not been in contact with a case in the particular infectious disease under investigation. In addition, this system will also issue text-based popup notifications to phone users if the number of contacts of one person (nearby potential contact) exceeds a certain threshold, say 500 or in a certain location.

### D. Research Question-3

*What model can both scale and preserve privacy?*

**FIGURE 10.**
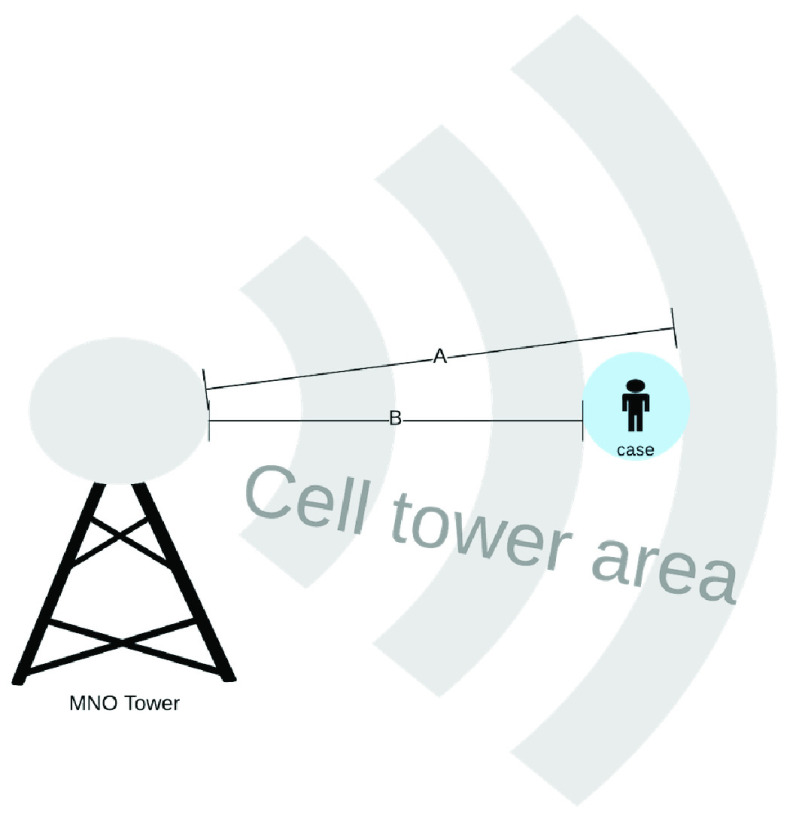
MNO accuracy of CDR triangulation [36].

The MNOs can track activity and movement of connected Subscriber Identity Modules (SIMs) when a powered on. It is possible to use the cell tower triangulation method for mobile device location determination to find the accuracy ranged between 50 and 300meters, which is the difference between A and B in Figure 10 [36]. Besides, the CDR information is held by telecommunications providers which are not censorship-resistant. Also the telecommunications call detail supported contact tracing can be abused if adequate measures are not in place. There are mechanisms to ensure the information provided are encrypted. The best use of this is for population-level mapping as the proximity requirements for COVID-19 make CDR data in-practicable.

Looking at Table 1, one can see that provider-facing applications are both challenging to scale and are low on privacy ranking as the collected information is processed both by the service provider and by the government. The use of call centre preserves privacy but relies on memory recall of the patient and their cooperation. Furthermore, since it requires human interaction, it is not scalable. Provider tracker and call center are solutions best suited for symptoms tracking. Health authorities also use video surveillance are which maybe expensive to scale and do not preserve privacy by default.

The citizen mobile application utilizes peer-to-peer communications amongst each other, but connect to the blockchain through the internet as in figure 6. Our model eliminates the need for follow-up during the 14 days of a contact quarantine as proximity notification at an individual level is automatically implemented by the smart contract. The citizen-facing mobile application-facing is scalable but may suffer from boycotts due to privacy concerns as seen in the case in India. These concerns are hinged on all data being stored and managed by the government. All current implementation of citizen application has data centrally managed by the government. The RFID chips, on the other hand, are often implemented with a centralized server. None of the current implementation to our knowledge helps preserve the privacy of the parties while ensuring scalability. Our proposed system stores data on a blockchain log, but at an average cost of $0.34 per log. We believe this makes our proposed model and solution scalable.

It extends the current citizen and health worker applications by providing guarantees that the data stored is independent of government censorship. Our system does not also need off-chain storage because the data captured are small, and can be logged directly on the blockchain.

It also makes available options for citizens interested in tracking animals using RFID tags and end-point receivers. For the receiver to be completely censorship-resistant, its architecture must be open and made available for audit to guarantee that it is not sending information to an alternate server. However, this area of work is beyond the scope of this research.

An individual can optionally volunteer to allow their call detail information to be mapped to retrace their movement after they have tested positive. This will help with the accurate decontamination efforts, though [36] noted that accuracy of using call detail registry is between 50meters and 300meters. This location sensitivity can be visualized from a distance between A and B in Figure 10.

In our proposed model, the case information is not linkable on the blockchain, users who have met the contact criteria set on the blockchain smart contract will receive a notification on their application with the current status of yellow or green. What we describe will only work for citizen application users.

### E. Limitations

A key limitation of this study is that the COVID-19 pandemic is evolving and thus, very little scholarly articles have been published on the use of digital technologies for contact tracing. A few of the country-specific solutions are based on newspaper articles and blog posts. If a government mobile application is used, it can still be a source of centralization if not made open for audit. External systems are not censorship-resistant and can be a source of centralization. Our prototype smart contract did not implement security and other fine-grained solution required for production-grade smart contracts. However, these areas are not the focus of this study.

## VII. Conclusion

Contact tracing is among the many complementary strategies for reducing, halting, and reversing COVID-19 infection and deaths. In this article, we have reviewed strategies for digital-enabled contact tracing, technologies, usage, and network options. We found that most digital contact tracing strategies are either not scalable or do not preserve the patient’s privacy. We also found that current contact tracing measures do not consider moving objects.

We designed and presented a novel contact tracing system model using IoT and blockchain. We implemented three smart contracts as the prototype and simulated deployment and function calls. We show that our system can help citizens preserve their privacy while voluntarily participating in contact tracing and notification. We also found and presented the deployment and execution costs on the Ethereum blockchain.

Our model is equally novel because moving objects can be tracked using proof-of-concept RFID transceiver and storing the information on the blockchain to preserve the owner’s privacy until required. This solution will help understand human connectivity and model infection spread networks. It can also be used to identify super spreading persons, animals, events, places, or objects. Furthermore, it can aid the development and implementation of public policies to control the spread of COVID-19 and prepare for any future epidemic and pandemic. Our future work will be to implement the RFID solution along with either existing frontend applications or a new frontend application. Our future work will also seek to reduce the cost of scaling this solution.
